# Paper‐Origami‐Based Multiplexed Malaria Diagnostics from Whole Blood

**DOI:** 10.1002/anie.201606060

**Published:** 2016-08-24

**Authors:** Gaolian Xu, Debbie Nolder, Julien Reboud, Mary C. Oguike, Donelly A. van Schalkwyk, Colin J. Sutherland, Jonathan M. Cooper

**Affiliations:** ^1^ Division of Biomedical Engineering, School of EngineeringUniversity of GlasgowOakfield AvenueGlasgowG12 8LTUK; ^2^ Public Health England Malaria Reference Laboratory (PHE MRL)Faculty of Infectious & Tropical DiseasesLondon School of Hygiene & Tropical MedicineUK; ^3^ Dept of Immunology & InfectionFaculty of Infectious & Tropical DiseasesLondon School of Hygiene & Tropical MedicineUK

**Keywords:** diagnostics, malaria, microfluidics, nucleic acid based test, paper origami

## Abstract

We demonstrate, for the first time, the multiplexed determination of microbial species from whole blood using the paper‐folding technique of origami to enable the sequential steps of DNA extraction, loop‐mediated isothermal amplification (LAMP), and array‐based fluorescence detection. A low‐cost handheld flashlight reveals the presence of the final DNA amplicon to the naked eye, providing a “sample‐to‐answer” diagnosis from a finger‐prick volume of human blood, within 45 min, with minimal user intervention. To demonstrate the method, we showed the identification of three species of Plasmodium, analyzing 80 patient samples benchmarked against the gold‐standard polymerase chain reaction (PCR) assay in an operator‐blinded study. We also show that the test retains its diagnostic accuracy when using stored or fixed reference samples.

Nucleic acid based tests (NATs) offer the promise of microbial diagnostics, determining either the species present or characteristics of the pathogen, such as drug resistance. The gold‐standard assay used in many reference laboratories is based upon a polymerase chain reaction (PCR) amplification—a technology which achieves high sensitivities but which also requires trained staff and external power. In contrast, identification of microbial species in resource‐limited environments requires low cost, simple tests that do not need external or fixed power supplies. One example where such a simple low‐cost test could transform outcomes is in malaria diagnosis, where species identification directly informs patient treatment.

Classical malaria diagnosis involves a blood smear followed by microscopy, which, although simple, does not provide the required sensitivity and only enables species specific information in the hands of trained experts.^1^ New approaches will be required to tackle the disease, where asymptomatic individuals commonly harbor the disease at levels that are below the sensitivity of microscopy (<100 parasites/μL).^2^ Nucleic acid based tests (NATs) offer the promise of achieving such high sensitivities (1 parasite/μL) with excellent specificity,^3^ enabling healthcare professionals to inform treatment.^4^

As many people living at risk of malaria infection have no access to diagnosis, presumptive treatment of all febrile patients as if they were malaria cases is a common practice, which has become a serious problem, especially in sub‐Saharan Africa.^5^ Currently, the most widely adopted NAT method in infectious disease diagnosis is still PCR, although the reliance on thermocycling has proven a barrier to its implementation in low‐resource settings.^6^ LAMP has emerged as a low‐cost alternative,^7^ simplifying hardware requirements whilst enabling visual detection.^8^ Although amenable to multiplexing, the high number of primers required (up to six per target)^9^ restricts the number of targets that tests can detect in one reaction. A commercial *Plasmodium* genus LAMP test is available, but the system requires a multi‐step DNA extraction based on the PURE methodology, carried out on a bench‐top instrument. Alternatively, lateral flow tests, commonly referred to as “rapid diagnostic tests” (RDT), also exist,^10^ but their sensitivity is poor.^11^

Herein, we show a new capillary‐flow platform that combines ease‐of‐use and low‐cost with the sensitivity of LAMP, into a multiplexed three taxon‐specific test plus a control. We overcome the difficulties linked to sample preparation and multiplexing using capillary wicking and paper‐folding origami techniques to distribute fluids both vertically and laterally.^12^, ^13^

Previously paper microfluidics (see Review^14^) has enabled single units of a NAT, such as DNA extraction,^15^ DNA isothermal amplification,^16^ which have been integrated into a manual “machine”^17^ with hybridization‐based DNA detection.^18^ We now integrate all the required steps into a single device to detect *Plasmodium falciparum*, *Plasmodium vivax*,^19^ and *Plasmodium* pan directly from a finger‐prick volume of whole blood within an operator‐blinded study (Figure 1).

**Figure 1:**
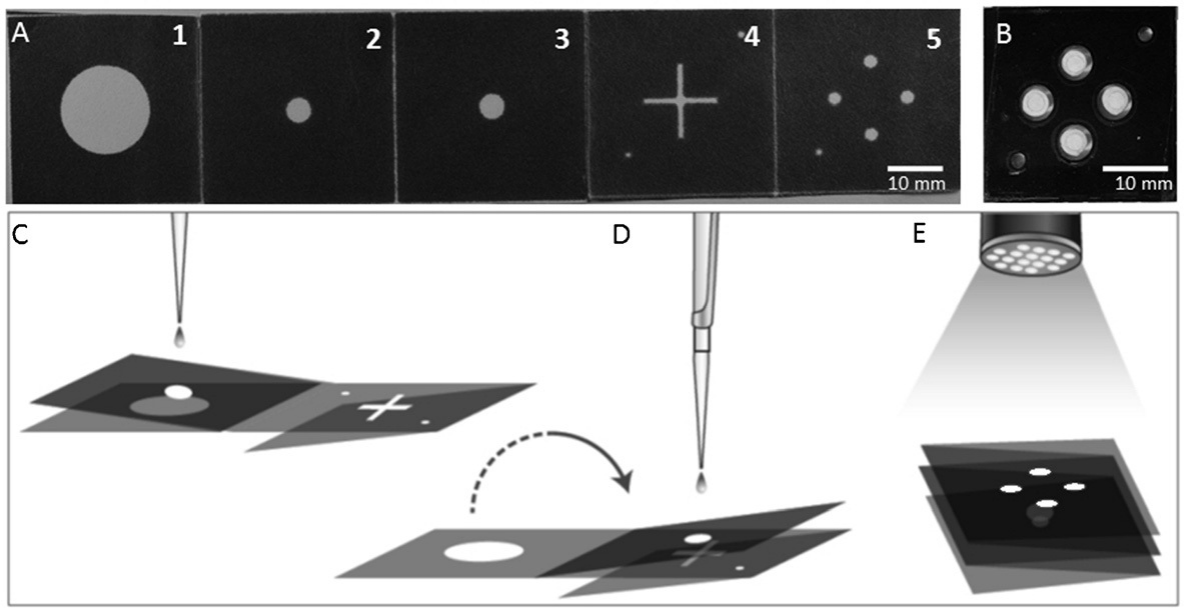
Paper‐based multiplexed LAMP detection of malaria in blood. A) Foldable paper devices: Dark areas are printed with hydrophobic wax. The device consists of five panels (1–5) folding onto each other, and a plastic cover for LAMP processing to avoid evaporation (B). The design also incorporates alignment marks on two corners (in the bottom left and top right corners) to assign the results. C) Illustrates the extraction process. Panels 2 and 3 are folded together and onto Panel 1. The sample is dispensed onto the device (panel 3) and extracted using capillary flows vertically (flow of liquid from Panel 3 to Panel 1). By folding the device (flipping the Panel 2–3 fold onto Panels 4 and 5), the sample is transferred to the LAMP spots (D) where the reaction is carried out. The signal is read out using a UV flashlight (365 nm; E).

Often in‐field testing cannot be performed for logistical reasons, and retrospective diagnosis is required. Testing of archived blood samples is also important for epidemiologists to re‐visit reference samples or to analyze historical data sets.^20^ We therefore show that we can identify parasites in preserved samples of frozen whole blood, as thick and thin fixed smear samples on glass slides, and as whole blood dried onto paper.

The fabrication of the device using wax printing,^21^ as well as the operating steps, shown in Figure 1 and Figure 2, are all detailed in the Supporting Information. After addition of the sample onto the device (Panel 3, Figure 2 A), the paper was folded (structure S1, Figure 2 B) to enable the first steps of the assay, involving cell lysis and DNA extraction, to yield purified DNA (Figure 2 B–D) on the glass‐fiber paper. To transfer the DNA from the extraction panel to the amplification panel, the fold S1 is flipped on the opposite side (Figure 2 E), allowing elution (Panels 4–5 of Figure 2 A and F). Supporting Information Figure S1(A) illustrates the extraction process.

**Figure 2:**
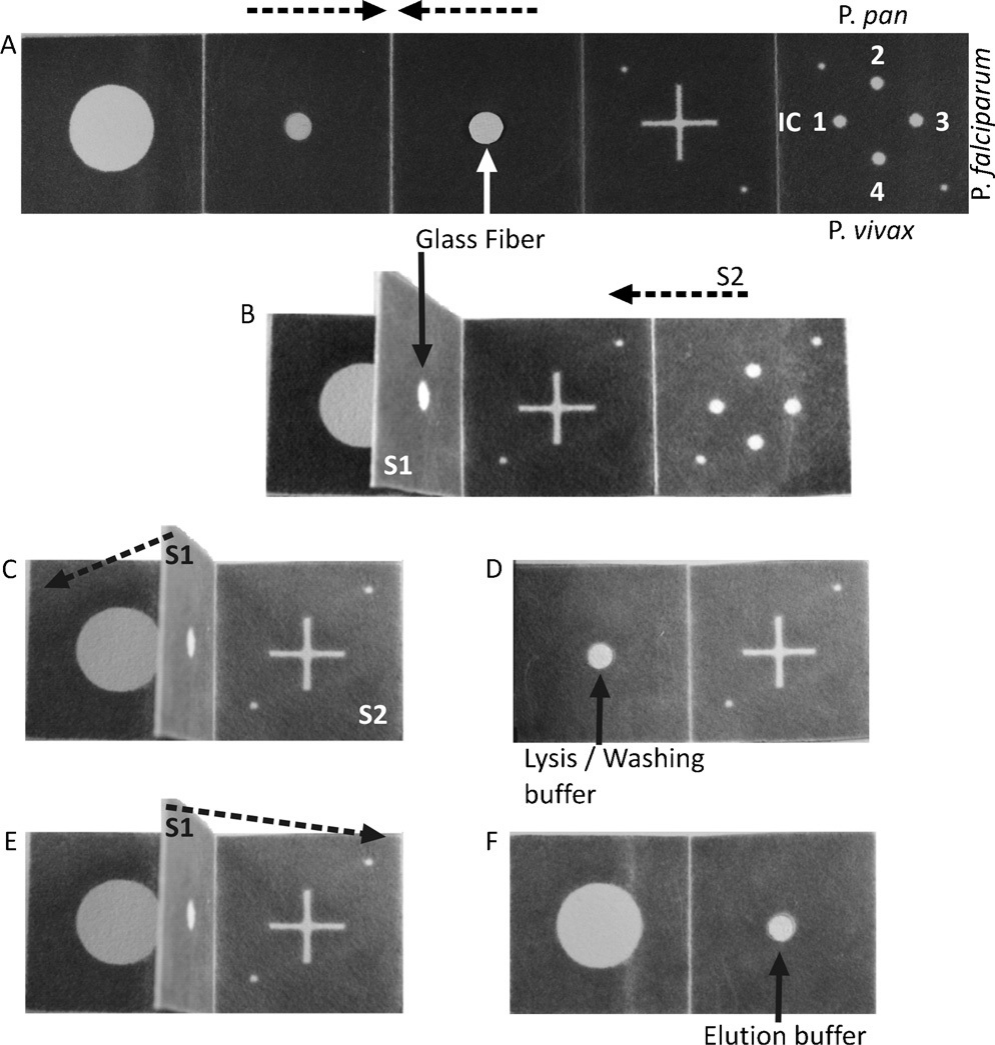
Paper‐folding steps for fluidic manipulation of assay steps. The broken arrows indicate folding direction. Panels numbered as in Figure 1. A) A hole from the center of the third Panel has a glass‐fiber disc onto which the sample is dispensed; Numbers in the last panel indicate the different reagents placed onto the four different spots for amplification of different species. 1. Internal control (IC); 2. *Plasmodium* pan; 3. *P. falciparum*; 4. *P. vivax*. B) The second/third Panel are folded and clamped to form structure 1 (S1); C) The fifth Panel is folded onto the back of the fourth frame to form structure 2 (S2); D) S1 is folded onto the first Panel before adding lysis/washing buffer for DNA extraction and purification; E–F) S1 is folded onto S2 for elution and elution buffer added.

Multiplexing analysis was enabled by using capillarity to guide the sample to four independent locations on the paper within hot wax printed channels, where species‐specific LAMP reagents were deposited (Figure 2 A, panel 5). The system was sealed by an acetate film to prevent evaporation during incubation (Figure 1 B and D) and amplification was carried out 63 °C for up to 45 min on a simple hotplate. The results of species‐specific LAMP were initially read‐out by the naked eye with a handheld UV lamp (365 nm; Figure 3 A).^8^

**Figure 3:**
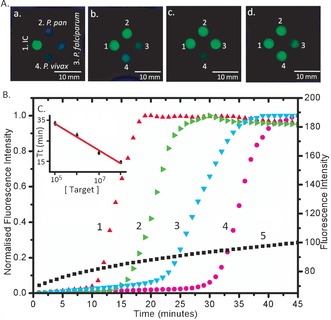
Results of multiplex LAMP amplification under UV light. Under UV excitation, green calcein emission occurs in the presence of pyrophosphate A) Numbers denote different species‐specific LAMP reaction. 1. Internal control (IC); 2. *Plasmodium* pan; 3. *P. falciparum*; 4. *P. vivax*. Letters denote the different positive results: a. 1 positive; b. 1 and 2 positive; c. 1, 2 and 3 positive; d. all positive. B) Real‐time amplification curve of *Plasmodium* pan LAMP with the 10‐fold serially diluted target (1–4, normalized real‐time amplification curves), and ddH_2_O as a negative control (5): 1. 10^8^ IU/mL (red up‐triangle); 2. 10^7^ IU/mL (green right‐triangle); 3. 10^6^ IU/mL (cyan down‐triangle); 4. 10^5^ IU/mL (magenta circle). 5. Negative control (black square: no target DNA). As the concentration increases, the amplification is initiated earlier, evidenced by the exponential increase in the fluorescence. C) Threshold time (defined as the time corresponding to 50 % of the maximum fluorescence intensity, Tt) as a function of target concentration. This Figure of merit is analogous to the cycle threshold (Ct) of real‐time PCR.^23^ Data is the average of three repeats and error bars represent the standard deviation. The data was fitted with linear regression (*R* ^2^=0.98).

We also showed that the technique was amenable to quantification (Figure 3 B,C). To test sensitivity, we used the WHO International Standard for *P. falciparum* DNA,^22^ which was serially diluted from 10 to 10^4^ times. The real‐time amplification curves (Figure 3 B) were normalized to 1 for ease of comparison and show that sensitivity down to 10^5^ IU/mL can be achieved within 35 min. As the copy number decreases, so the exponential phase of signal enhancement starts later (Figure 3 C) as a consequence of diffusion limited reaction kinetics. For highly infected samples, the time to detection can be significantly faster, down to 12 min for 10^8^ IU/mL. 80 fully characterized fresh (unfrozen) EDTA‐blood samples from the PHE MRL were then tested by origami‐LAMP in an operator‐blind experiment.

Our method showed high specificity and good sensitivity for identifying *Plasmodium* in blood samples (Table 1), when compared against the benchmark PCR.^3^, ^24^ Only for *P. falciparum* was sensitivity below 80 %, as a number of samples for this species were of low parasite density.

**Table 1:** The diagnostic accuracy for multiplex‐LAMP against reference laboratory diagnosis (using PCR as a benchmark).

	Origami LAMP			Sensitivity	Specificity
		Positive	Negative		
Benchmark PCR					
*P. falciparum*	Pos	11	7	61 %	98 %
	Neg	1	61			
					
*P. ovale* spp./ *P. malariae*	Pos	27	2	93 %	96 %
	Neg	2	49			
					
*P*. vivax	Pos	17	4	81 %	98 %
	Neg	1	58			
					
Any Plasmodium spp.	Pos	59	10	86 %	100 %
	Neg	0	11				

All samples were also tested with a commercially‐available LAMP kit for malaria (Eiken Chemical Company Ltd. (Japan)), which covers two of our three targets (*Plasmodium* pan and *P. falciparum*; Table 2). The specificity for *Plasmodium* pan and *P  falciparum* between our method and the LAMP kit, which deploys readout in a commercial turbidimeter, were above 98 %, while sensitivity was lower at 88 and 69 % respectively (Table 2).

**Table 2:** Species‐specific malaria diagnosis by commercial LAMP and origami‐LAMP.

	Origami LAMP			Sensitivity	Specificity
		Positive	Negative		
Commercial LAMP					
*P. falciparum*	Pos	11	5	69 %	98 %
	Neg	1	63			
					
Any Plasmodium spp.	Pos	60	8	88 %	100 %
	Neg	0	12				

Compared to the commercial LAMP kit, our platform has two advantages: origami LAMP is able to differentiate between *P. falciparum* and *P. vivax* infection, and correctly identify non‐vivax/ non‐falciparum, while also providing pan‐genus diagnosis as a single test. This has implications in guiding case management since these two species are associated with different treatments.^25^, ^26^ *P. falciparum* is more likely to progress to a severe illness than *P vivax*, while *P. vivax* requires treatment of the dormant form of the parasite. The two species also present different drug resistance profiles.^26^ The inclusion of an internal control is an important quality control element for ruling out false negatives due to test failure.

Our method is more sensitive than microscopy and showed a close coincidence with both the gold standard benchmark (PCR) and a commercial LAMP assay (>90 %, except for the PCR *P. pan* assay, 88 %—detailed calculations in Table S1). The origami LAMP test only failed to detect seven weakly positive samples. These included PCR‐positive individuals with negative blood films, and patients that had already commenced antimalarial treatment. Importantly, if we exclude these challenging samples from the test panel, the sensitivity increases to 100 % for *P. falciparum* and 95 % for *P. pan*, while coincidence with other methods reach above 93 % for all assays.

We also studied the analytical sensitivity of the technique using serially diluted cultured samples in whole blood, demonstrating detection down to 5 parasites/μl (a threshold below that of routine microscopy and below the clinical threshold at which symptoms of malaria occur, 500 parasites/μL^27^—see Methods and Figures S2‐3 in the Supporting Information). Clinically, this allows any user to consider that a negative test in a febrile patient will be indicative of the fact that malaria infection is not the cause of the fever, although the presence of a low density parasitaemia of *Plasmodium* spp. cannot be ruled out. Of particular importance is that the high specificity of our method, and thus low false positive ratio, gives the user confidence that a positive result truly indicates the presence of malaria.

To analyze samples retrospectively, we stored 4 blood samples under four different conditions (frozen, as thick and thin smears fixed in acetone, and dried). Table S2 shows that there is no difference between using frozen and fresh samples (Figure S4 shows the details of the images obtained), enabling retrospective analysis when testing in the field is not possible or in large cross‐sectional studies.^28^ As an additional feature of paper‐based devices for disease diagnostics, we noted that samples can be readily disposed of by incineration.^29^

## Supporting information

As a service to our authors and readers, this journal provides supporting information supplied by the authors. Such materials are peer reviewed and may be re‐organized for online delivery, but are not copy‐edited or typeset. Technical support issues arising from supporting information (other than missing files) should be addressed to the authors.

Supplementary
