# Supplementary material for: Paper‐Origami‐Based Multiplexed Malaria Diagnostics from Whole Blood
Source: Angew Chem Int Ed Engl. 2016 Aug 24;55(49):15250–3. doi: 10.1002/anie.201606060 (PMC5132111; doi:10.1002/anie.201606060)
Supplement: Supplementary file 1 — Supplementary [file ANIE-55-15250-s001.pdf]

Angewandte Chemie
Eine Zeitschrift der Gesellschaft Deutscher Chemiker

Supporting Information

Paper-Origami-Based Multiplexed Malaria Diagnostics from Whole Blood

*Gaolian Xu+, Debbie Nolder+, Julien Reboud, Mary C. Oguike, Donelly A. van Schalkwyk, Colin J. Sutherland, and Jonathan M. Cooper**

anie_201606060_sm_miscellaneous_information.pdf

Experimental Section

Samples: The extraction process was developed using cultured CMV genomic DNA (ATTCC, VR-538D) spiked in water at different concentrations. DNA recovery was analysed using real-time PCR (see Supplementary Material section below). Subsequently, anonymised human blood samples from the Public Health England (PHE) Malaria Reference Laboratory (MRL) were tested, each having been previously characterised to species-level by expert microscopy and standard molecular diagnostic protocols used in the MRL - this comprises a combination of species-specific nested PCR[1] and real time qPCR[2]. All samples were also tested with a commercially-available LAMP kit for malaria (Eiken Chemical Company Ltd. (Japan)), which covers two of our three targets (*Plasmodium* pan and *P. falciparum*). The analytical performance of the assay was studied utilizing a commercially available source of *P. falciparum* DNA – the WHO International Standard (WHO-IS).[3]

Sensitivity Analysis: parasite culture and sample preparation: Plasmodium falciparum parasites (3D7 strain) were cultured using a modified method of Trager and Jensen[4]. Briefly, parasites were grown in RPMI 1640 media supplemented with glucose (10 mM), Albumax II (0.5 g/L), L-glutamine (2 mM), hypoxanthine (147 µM), HEPES (25 mM), Na2HCO3 (25 mM), gentamicin (25 µg/mL), and 2% (v/v) human AB serum. Parasites were maintained daily in culture at 4% haematocrit using human A+ red cells and were synchronised with D-sorbitol (5% w/v) as described previously[5]. Infected RBCs were harvested, pelleted (centrifuged at 600 x g for 5 minutes) and diluted in whole blood to the required number of parasites per microliter, in order to test the sensitivity of the device. Synchronised ring stage parasites were used to mimic the likely parasite population present in *P.falciparum* infected human venous blood.

Multiplex LAMP system: We based our LAMP system on previously published primer sequences for *Plasmodium falciparum* specific,[6] *Plasmodium vivax* specific[7] and *Plasmodium* genus. A BCRA1 gene fragment was added into the sample and served as an internal control (IC).[8] All the primers were purchased from Eurofins Genomics (sequences listed in Table S3 below). The 20 µL malaria-specific LAMP mix contained 1.5 µM of the inner primers (FIP and BIP), 0.8 µM of the loop primers (LPF and LPR), and the outer primers F3 and B3 at 0.05 µM and 0.2 µM, respectively. The primer concentrations for the internal control were 1 µM (FIP/BIP), 0.5 µM (LPF/LPR), and 0.05 µM (F3/B3). For the internal control, 8 units Gsp SSD DNA polymerase (OptiGene) was added to the mix, whereas 12 units was used for test reactions. The mixes also contained 0.4 mM of each dNTPs (Sigma), 4.0 mM of $MgSO_4$, 50 mM Tris-HCl (pH 8.1), 30 mM KCl, 30 mM $(NH_4)_2SO_4$, 0.1% Triton X-100, 1 M of Betaine, 25 µM Calcein (Sigma), and 500 µM $MnCl_2$.The multiplex LAMP reactions were performed at 63 °C for 45 minutes. The results were read out with a handheld-UV lamp (wavelength 365 nm), revealing the precipitation of manganese using Calcein as an indicator for the presence of Ca^{2+}.[9]

Paper devices: The fabrication process was performed without specialised facilities or a clean room by simply using a wax printer and a hot plate (the latter can also be used to carry out the LAMP reaction). Each device contained three components, namely: a filter paper based fluidic device where the liquid was constrained by printed hydrophobic wax; a single sided adhesive acetate film sealed plastic plate with 4 glass fibre spots (diameter 3mm), which form chambers for LAMP reaction (Figure 1B); and one glass fiber spot (Figure 2A) (GFF, Whatman)[10] with diameter 4 mm for absorbing nucleic acids from the sample in the presence of high concentration of the chaotropic agent, guanidine thiocyanate (GuSCN).[11]

In order to assemble the device, the filter paper was first printed with black wax using an office printer (XeroxColorQube 8570),[12,13] then heated at 120 °C for 1 min on the hot plate to melt the printed wax, which diffused through the paper to form the same hydrophobic pattern of channels and vias on both sides. Subsequently the glass fiber spots were manually positioned so that appropriate folding manipulations allowed the easy transfer of reagents and sample. Finally the device was then sealed using two acetate films (MicroAmp® Optical Adhesive Film, Thermo Scientific), or cling film, preventing liquid evaporation during the amplification.

DNA extraction from blood: 20 µl of blood was mixed with 150 µl lysis buffer (L6 – 1.20 g/ml of GuSCN, 0.1 M Tris hydrochloride, 0.04 M EDTA, adjusted with NaOH to pH 8.0, 26 mg/ml Triton X-100)[11], before heating at 95 °C for 5 minutes. The lysis buffer permeated the glass fiber and was absorbed by capillary action into the surrounding blotting paper (Figure 1A, panel 1). The DNA was drawn in this flow and captured by glass fiber (Figure 1A, panel 1). Subsequently, 100 µl washing buffer containing 70% ethanol and 30 mM NaOAc was used to wash cell residues away. After washing, 40 µl elution buffer (10 mM TE buffer, pH 8.0) was used to elute the nucleic acid from the glass fiber and onto the printed LAMP reagents (Figure 1A, S2). We characterized the extraction process using artificially-spiked samples, showing a recovery above 60% (Figure S1).

Reference & Stored Samples: To establish the origami test's ability to detect parasite DNA in stored blood, we split 4 fresh blood samples from the PHE MRL into 5 aliquots and stored each of these under different conditions: (1) frozen at -20°C, (2) thick smears and (3) thin smears on glass slides and fixed with acetone, (4) air dried at room temperature on filter paper (Whatman 3 M filter paper), and (5) no storage (these samples were tested directly as a reference for this study). We then tested each of these samples in duplicate on the origami platform and using the qPCR standard assay to confirm their status. The samples were processed following the same procedures as outlined above for fresh samples, with the addition of a recovery step for fixed and dried samples, using 100 µl of 0.4% SDS buffer.

Quantification: The LAMP reaction was quantified in real-time using a fluorescence microscope (Axio Scope A1, Zeiss) with a 10x objective and a FITC filter set (490 nm excitation, 515 nm emission). Data was collected every minute for 45 minutes (Hamamatsu) and quantified using the Wasabi software (Hamamatsu Photonics).

Statistical analysis: For each pairwise comparison between the index test (ORIGAMI LAMP) and the reference (BENCHMARK PCR), the null hypothesis of no difference in performance between the 2 tests was retained for P values of ≥ 0.05, determined by the McNemar test. Confidence intervals (CIs) were calculated on the basis of the binomial distribution using the STATA 14.1 statistical package (StataCorp LP, Texas, USA).

Supplementary Material.

Characterisation of Extraction: We characterized the extraction process using buffer spiked with human Cytomegalovirus (CMV) targets available in our laboratory.[14] We then performed a real-time PCR analysis of the recovered DNA to establish the efficiency of the process. The PCR system contained : 1 µM forward primer (5'-AAC CCG GCA AGA TTT CTA ACG-3'), 1 µM reverser primer (5'-ATT CTG TGG GTC TGC GAC TCA-3'), and 0.5 µM probe (5'-CTA GTC ATC GAC GGT GCA CAT CGG C-3'), 7.5 µl Brilliant III Ultra-Fast QPCR Master Mix (Agilent Technologies Inc.), 2 µl target with total volume 15 µl. The PCR was performed on an ABI 7500 as follows: 94 °C for 3 min; 40 cycles of 94 °C for 12 s, 60 °C for 30 s. The CMV genomic DNA was serially diluted (1:5) from 12.5 ng/µL (test by BioTek). The diluted DNA was put through the extraction process on the paper device as detailed above. After elution, 1 µl of liquid was analysed by real-time PCR to quantify the amount of DNA, compared to the original dilutions. The DNA recovery rates for different concentrations were between 60-70% (Figure S1B).

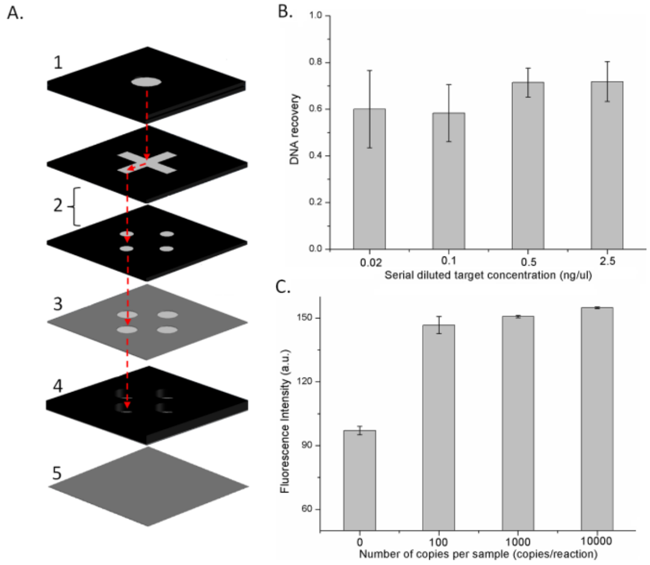

Figure S1 (**A**): Illustration of the pathway taken by DNA molecule through the folded device during elution. 1. Structure S1 clamped with glass fibre (white); 2. Structure S2 (see Figures 1&2); 3. Acetate film with filter paper spots (white); 4. Plastic plate with holes (grey); 5. Acetate film. The red dotted line denotes the route of DNA movement at dilution step; (**B**). The DNA recovery rate (1 denotes a perfect recovery without loss) obtained by real-time PCR with 1:5 serial diluted CMV genomic DNA form 12.5 ng/µl. (**C**) Fluorescence intensity of positive and negative samples obtained from the internal control (IC). Error bars represent the standard deviation.

Table S1: Coincidence with benchmark methods.

Test Method	Plasmodium species	Coincidence with Origami LAMP	
PCR	*P.falciparum*	72/80	90%
	P.ovale/P.malariae	76/80	95%
	P.Vivax	75/80	94%
	Any Plasmodium spp.	70/80	88%
Commercial LAMP	*P.falciparum*	74/80	93%
	Any Plasmodium spp.	72/80	90%

List of the 7 *P. falciparum* samples not detected by Origami but detected by PCR: 2 National EQA (NEQAS) test samples at densities below the microscopy threshold. 2 microscopy-negative sub-patent infections, 1 chronic infection from a recent refugee, with only scanty gametocytes on microscopy, 2 UK residents of African origin who took anti-malarial drugs in-country before returning to UK for further diagnosis and treatment.

Analytical sensitivity analysis: We studied the analytical sensitivity of our method through serial dilutions of cultured *P. falciparum* into uninfected whole blood, quantified through microscopy. Figure S2 below shows that the origami device was able to detect concentrations down to 5 parasites/µl by simple visual observation. We further quantified the intensity of color green in each spot of Figure S2 and normalised these to the controls (subtracted the intensity of the negative spot and then divided by the intensity of the positive control – IC), such that a value around 1 indicated a positive spot, while values around 0 indicated a negative spot. Using this methodology, the sensitivity of the origami test was also at 5 parasites/µl (Figure S3), using a t-test to differentiate from the negative control (0 parasites/µl – p-value of 10^{-5} for 5 parasites/µl and 3.10^{-3} for 10 parasites/µl)

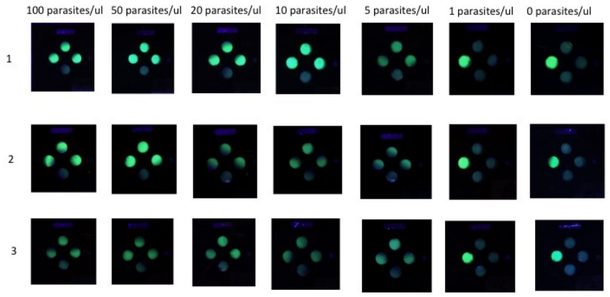

Figure S2 Analytical sensitivity: Pictures of the devices after origami LAMP of diluted *P. falciparum* infected RBCs into whole blood. Columns are for different concentrations (left to right: 100, 50, 20, 0, 5, 1, 0 parasites/µl). Rows are 3 repeats of the same concentration. The pictures were taken under UV illumination. The same orientation is used as in the main text (right-hand spot is IC, top spot *P. pan*, left spot *Pf*, bottom spot *Pv*, negative control here).

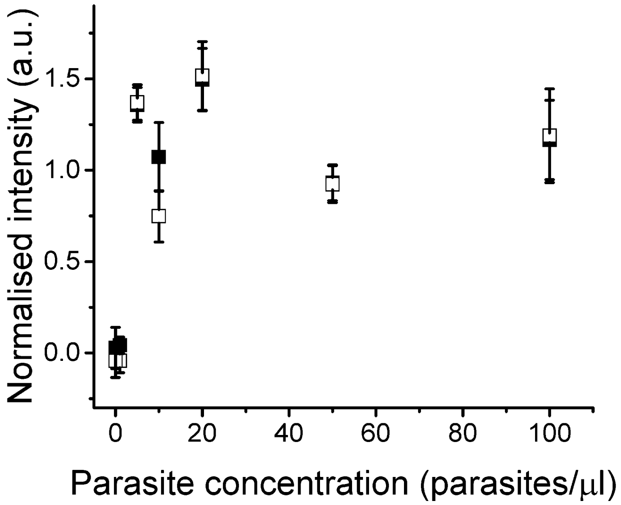

Figure S3. Quantification of the analytical sensitivity. The intensity for the colour green in each spot was quantified and normalized to the positive (IC) and negative (Pv spot) controls (presented in arbitrary units a.u.). Black squares are the values for the Ppan spot, white square is for the Pf spot.

Sample storage results

Table S2. Comparison of test performance on preserved blood samples

	Fresh		Frozen		Fixed (thin smear)		Fixed (thick smear)		Dried		qPCR
A	Pf	Pf	Pf	Pf	Pf	Pf	Pf	Pf	Pf	Pf	Pf
B	Pf	Pf	Pf	Pf	Pf	Pf	Pf	Pf	Pf	Pf	Pf
C	Neg	Neg	Neg	Neg	Neg	Neg	Neg	Neg	Neg	Neg	Neg
D	Neg	Neg	Neg	Neg	Neg	Neg	Neg	Neg	Neg	Neg	Neg

Results are benchmarked using the standard qPCR assay.
(Pf – *P. falciparum*; Neg – Neg – negative result)

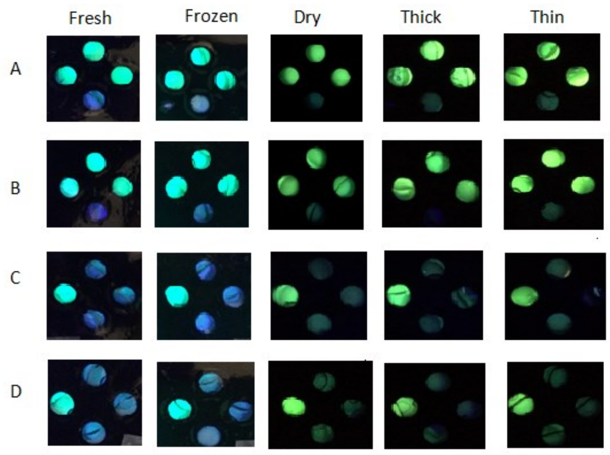

Figure S4. Pictures of each test result for different storage conditions (tabulated in Table S2).

Additional References.

[1] G. Snounou, S. Viriyakosol, X. P. Zhu, W. Jarra, L. Pinheiro, V. E. do Rosario, S. Thaithong, K. N. Brown, *Molecular and Biochemical Parasitology* **1993**, *61*, 315–320.

[2] S. E. Shokoples, M. Ndao, K. Kowalewska-Grochowska, S. K. Yanow, *J. Clin. Microbiol.* **2009**, *47*, 975–980.

[3] D. J. Padley, A. B. Heath, C. Sutherland, P. L. Chiodini, S. A. Baylis, $author firstName $author.lastName, *Malaria Journal* **2008**, *7*, 139.

[4] W. Trager, J. Jensen, *Science* **1976**, *193*, 673–675.

[5] C. Lambros, J. P. Vanderberg, *The Journal of Parasitology* **1979**, *65*, 418–420.

[6] S. D. Polley, I. J. González, D. Mohamed, R. Daly, K. Bowers, J. Watson, E. Mewse, M. Armstrong, C. Gray, M. D. Perkins, et al., *J Infect Dis.* **2013**, *208*, 637–644.

[7] E.-T. Han, R. Watanabe, J. Sattabongkot, B. Khuntirat, J. Sirichaisinthop, H. Iriko, L. Jin, S. Takeo, T. Tsuboi, *Journal of Clinical Microbiology* **2007**, *45*, 2521–2528.

[8] N. A. Tanner, Z. Yinhua, E. Thomas C, *BioTechniques.* **2012**, *53*, 81–89.

[9] N. Tomita, Y. Mori, H. Kanda, T. Notomi, *Nat. Protocols* **2008**, *3*, 877–882.

[10] M. G. Weinbauer, I. Fritz, D. F. Wenderoth, M. G. Höfle, *Applied and Environmental Microbiology* **2002**, *68*, 1082–1087.

[11] R. Boom, C. J. Sol, M. M. Salimans, C. L. Jansen, P. M. Wertheim-van Dillen, J. van der Noordaa, *Journal of Clinical Microbiology* **1990**, *28*, 495–503.

[12] E. Carrilho, A. W. Martinez, G. M. Whitesides, *Anal. Chem.* **2009**, *81*, 7091–7095.

[13] Y. Lu, W. Shi, L. Jiang, J. Qin, B. Lin, *ELECTROPHORESIS* **2009**, *30*, 1497–1500.

[14] R. N. Gunson, A. R. Maclean, S. J. Shepherd, W. F. Carman, *J. Clin. Microbiol.* **2009**, *47*, 765–770.

.

Table S3. Primer sequences for multiplex-LAMP reactions

Species	Oligo name	Sequence
Plasmodium. pan	PANF3	TCGCTTCTAACGGTGAAC
	PANB3	AATTGATAGTATCAGCTATCCATAG
	PANLPF	TGGACGTAACCTCCAGGC
	PANLPR	CACTATACCTTACCAATCTATTTGAACTTG
	PANFIP	GGTGGAACACATTGTTTCATTTGATCTCATTCCAATGGAACCTTG
	PANBIP	GTTTGCTTCTAACATTCCACTTGCCCGTTTTGACCGGTCATT
P. falciparum	PFF3	CTCCATGTCGTCTCATCGC
	PFB3	AACATTTTTTAGTCCCATGCTAA
	PFLPF	GTTGAGATGGAAACAGCCGG
	PFLPR	CGGTGTGTACAAGGCAACAA
	PFFIP	ACYCAGTATATTGATATTGCGTGACAGCCTTGCAATAAATAATATCTAGC
	PFBIP	AACTCCAGGCGTTAACCTGTAATGATCTTTACGTTAAGGGC
P. vivax	PVF3	GGAATGATGGGAATTTAAAACCT
	PVB3	ACGAAGTATCAGTTATGTGGAT
	PVLPF	GCTGCTGGCACCAGACTT
	PVLPR	AGTTGAATTTCAAAGAATCG
	PVFIP	CTATTGGAGCTGGAATTACCGCTCCCAAAACTCAATTGGAGG
	PVBIP	AATTGTTGCAGTTAAAACGCTCGTAAGCTAGAAGCGTTGCT
BCRA1	BCRA1F3	TCCTTGAACTTTGGTCTCC
	BCRA1B3	CAGTTCATAAAGGAATTGATAGC
	BCRA1LPF	AGAACCAGAGGCCAGGCGAG
	BCRA1LPR	AGGCAGATAGGCTTAGACTCAA
	BCRA1FIP	ATCCCCAGTCTGTGAAATTGGGCAAAATGCTGGGATTATAGATGT
	BCRA1BIP	GCAGCAGAAAGATTATTAACTTGGG-CAGTTGGTAAGTAAATGGAAGA
